# Supplementary figures and images for: Comparative Metabolomic Profiling of L-Histidine and NEFA Treatments in Bovine Mammary Epithelial Cells
Source: Animals (Basel). 2024 Mar 29;14(7):1045. doi: 10.3390/ani14071045 (PMC11010852; doi:10.3390/ani14071045)

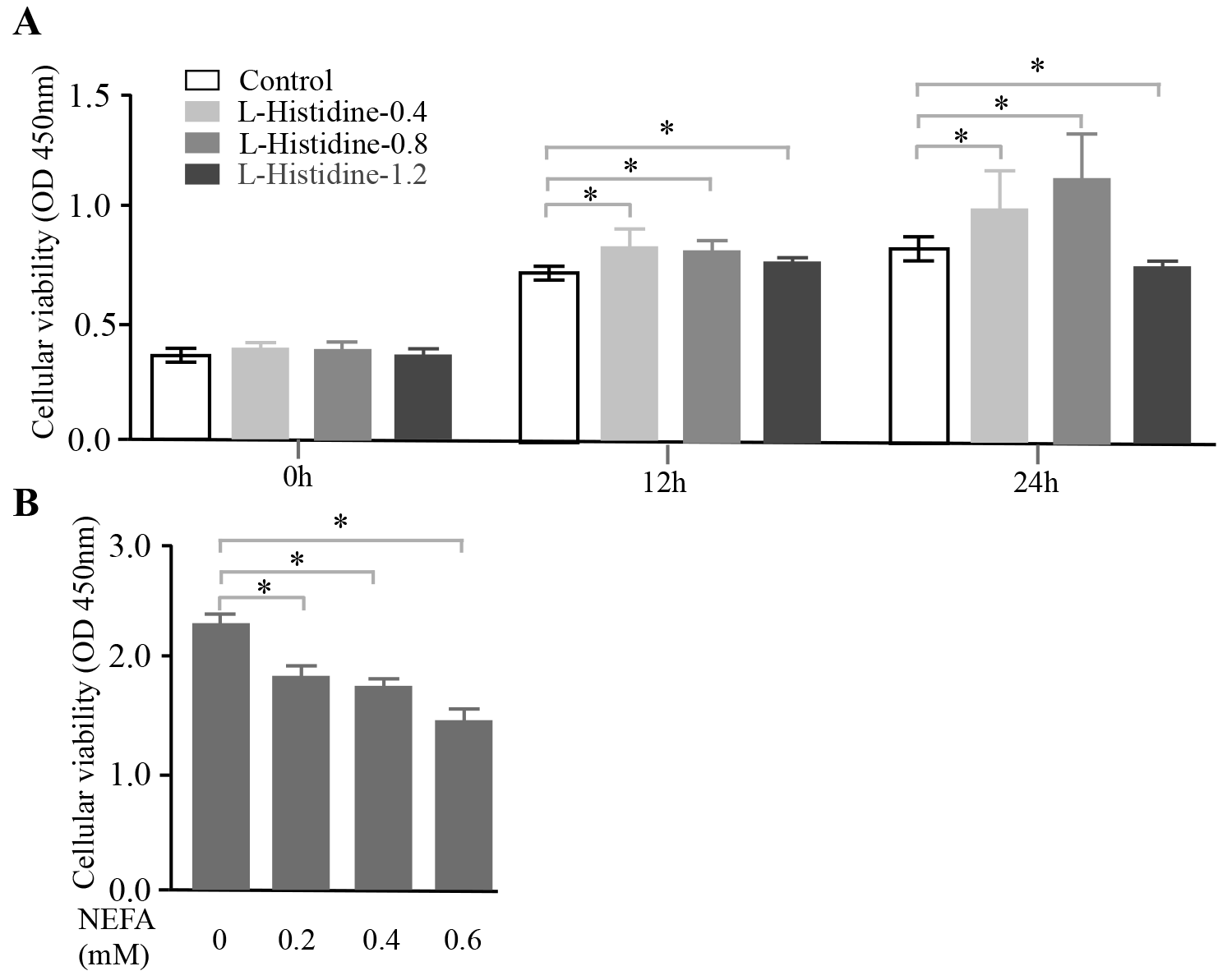

Supplement: Supplementary file 1 [file animals-14-01045-s001.zip › Figure S1 The effects of NEFA and histidine on the viability of BMECs.tif]
